# Bioethics at the intersection of politics, society, and healthcare: the significance of media debate analyses

**DOI:** 10.1186/s12910-025-01233-1

**Published:** 2025-07-04

**Authors:** Niklas Ellerich-Groppe, Bettina M. Zimmermann

**Affiliations:** 1 Division of Ethics in Medicine, Department for Health Services Research, Carl von Ossietzky Universität Oldenburg, Oldenburg, Germany; 2 Institute of History and Ethics in Medicine, TUM School of Medicine & Health, Technical University of Munich, Munich, Germany; 3 Institute of Philosophy, Faculty of Humanities, and Multidisciplinary Center for Infectious Diseases, University of Bern, Bern, Switzerland

**Keywords:** Empirical bioethics, Media discourse, Biomedical ethics, Methodology, Public debate, Ethical analysis

## Abstract

Since the “empirical turn” in bioethics, empirical inquiries have gained momentum in bioethical research. However, the relevance of the systematic analysis of media debates for empirical bioethics and the corresponding methodological requirements have so far been rather underexplored. Thus, the existing approaches are methodologically heterogeneous and their significance for bioethical inquiries has not been systematically discussed. In this paper, we provide a critical reflection on the significance of media debate analyses for bioethics, which lies in the possibility of investigating the unique intersection of bioethics, politics, society, and healthcare. Through a rapid scoping review, we outline the characteristics of published media debate analyses and show the heterogeneity of the field in terms of methodologies, academic disciplines, and topics covered. We identify four levels on which these publications contribute to bioethics research: (1) by providing a descriptive empirical context; (2) by describing ethical aspects of a health topic; (3) by identifying and evaluating moral problems; and (4) by providing an ethical evaluation of media debates. Based on this, we outline basic methodological requirements, address the limitations of media debate analyses for bioethics, and indicate recent and future advancements in the field.

**Supplementary Information:**

The online version contains supplementary material available at 10.1186/s12910-025-01233-1.

## Introduction

Since the ‘empirical turn’ in bioethics, empirical inquiries have gained momentum in the field [1–3], leading to the use of diverse and evolving methodologies [4]. Thus, semi-structured interviews, focus groups, or surveys with patients, caregivers, healthcare professionals, other experts, or a specific population have been widely applied in empirically informed bioethics. Various forms of content analyses are conducted in the contexts of scholarly literature reviews or law and policy reviews. Furthermore, media debates are increasingly getting attention as an empirical field in bioethics. However, these references are often made in rather unsystematic ways, for instance by using illustrative examples from the media discourse to set the stage for an ethical analysis or to underline an argument [5]. And even though systematic, methodologically reflected approaches inspired by social science research are increasingly used, the concrete potential and significance of media debate analyses for bioethics have so far been rather underexplored and require clarification. Why is it worth examining media debates from a bioethical perspective? What purposes do they serve for bioethical research? What methodological considerations are important to render these analyses significant for bioethics?

This paper aims to critically discuss these questions. We begin by highlighting the societal, practical, and ethical significance of media debates for bioethics. We then explore and systematize the field of media debate analyses in bioethics through a rapid scoping review and identify four levels on which analyses of media debates have contributed to bioethical inquiries so far. Finally, we discuss the basic methodological requirements of media debate analyses in bioethics, acknowledge the limitations of such investigations, raise open methodological questions, and set an agenda for future research in this area.

## The threefold significance of public media debate analyses for bioethics

Many salient issues in bioethics are also subjects of media debates, e.g., technological innovations in healthcare, health crises such as epidemics, or health policies. The following analysis applies a basic understanding of “media debates”, that hinges on three conditions: First, these debates take place in *news media* (i.e., newspaper, radio, television; sometimes also called legacy media) or *social media*. Indeed, the specific outlets, logic, relevant players and functionalities of news media differ considerably from social media, e.g., regarding possibilities for participation and gatekeepers. Still, they both serve as health information channels and arenas for public debates [6]. Second, media debates offer the possibility to communicate a variety of stances regarding a specific topic and foster an (indirect or direct) *exchange* of opinions as well as political and moral arguments. Consequently, we do not understand unidirectional websites, e.g., certain health information platforms or corporate websites, to be part of media debates. Rather, the variety of stances and the exchange on a specific topic usually presuppose different contributions that refer in some way to other contributions on this subject area. A single newspaper article would not be considered a debate in this sense, but the collective of various media contributions on a specific topic would. Third, our understanding of media debates presupposes *publicity*. Thus, we do not see communication in private social media groups as part of media debates, because they are not accessible to the broader public. As we will show, investigating such media debates has a threefold significance for bioethics.

### Societal significance

Media debates have inherent societal significance, particularly in democratic societies. Both traditional mass media and social media set the agenda of public debates and provide arenas for public debates [6, 7], fostering political deliberation and opinion formation. In this context, the political orientation of media corporations can influence media coverage, even though a recent international empirical investigation on an economic crisis indicates that national framings influence the plurality of media reporting more than political ideology [8].

Additionally, journalist-edited media fulfil an important control function in monitoring the activities of governments and other societal elites [9]. The importance of journalist-edited news media increases in times of crisis, as the COVID-19 pandemic has illustrated [10, 11]. As such, media debate analyses paint an important picture of whether and how people are exposed to bioethical issues and may contribute to raising awareness and shaping public opinion on these matters [12]. Because analyses of media debates allow insights into societal and political structures [13], they contribute to a better understanding of the societal context in which bioethical questions occur and give an indication of the relevance these questions have on the public agenda.

### Practical significance

Even though the concrete effects of media debates are multi-faceted and context-dependent [14], media debates can have practical consequences on decisions and actions of professionals, decision-makers and other relevant stakeholders in healthcare and health policy– depending on how health issues are discussed in these debates [15]. This may range from decisions on the health policy level to individual decisions in clinical practice and the acceptance of public health measures [16]. For example, the media coverage of COVID-19 contact tracing apps and how these technologies were presented in the media may have shaped how individuals accepted these apps as valuable contributions to combating the pandemic [17, 18]. These effects of media debates on health practice render their analysis relevant for bioethical investigation, since they can help to understand important factors around the application of bioethics.

### Significance for ethical theory

In addition to their significance for society and practice, we further argue for a specific *ethical* significance of media debates, considering their relevance for the identification of moral problems, descriptive ethics in general, and normative ethical arguments.

#### Identification of moral problems

Media debates can be relevant for the identification of moral problems in two respects: First, a media debate analysis brings together the political, practical, and ethical dimensions of a bioethical issue. Hence, it enhances the understanding of the societal and practical relevance of ethical aspects in a specific context and can show where certain moral norms and values are *not* fulfilled in practice. In an empirical investigation, these moral problems in the media debate can be carved out and evaluated afterwards. This involves both the discovery of previously unrecognized moral problems and the *re*-discovery of so far neglected moral problems. For example, Gerhards and colleagues analysed online forums to identify moral issues in live-in care [19], drawing attention to moral problems that have received only little consideration in bioethics so far.

Second, moral problems become not only apparent *in* media debates but also occur *due to* the media. Hence, in the analysis of media debates, it is also possible to reveal moral problems that emerge due to the way media present health topics. For example, the portrayal of older adults in media debates surrounding the COVID-19 pandemic may have reinforced age-related stereotypes and contributed to potential discrimination [20]. However, this moral problem can only be reliably identified through an empirical investigation of the media debate.

#### Descriptive ethics: a reflection of prevailing morality

Furthermore, media debates are significant for *descriptive ethics* in general since their analysis allows insights into the socially prevailing, dominant values, norms, and moral convictions. Analysing media debates in this regard provides a relevant overview of the moral landscape of a specific topic as discussed within a society and point to potential overemphasis or gaps, even though these aspects are often neither explicitly nor unambiguously reflected in media coverage. For example, public debates on climate change are pervaded with different moral norms and values that are sometimes only hidden in abstract references to climate protection [21]. A media debate analysis can help uncover colliding, unrealized, or neglected values, norms, and moral convictions. Hence, media debate analyses can be a relevant starting point for further ethical analyses and, thus, represent a “Lay of the Land” study for bioethics [22].

#### Normative ethical arguments

Finally, media debates are significant for *ethical arguments* in two respects. First, this concerns the *composition* of ethical arguments. For an argument to be correct, all premises, including empirical ones, must be valid [23]. This means that empirical premises should align with empirical findings and acknowledge empirical uncertainty or incomplete evidence, if applicable. When media debate content serves as an empirical premise in an ethical argument, a systematic media debate analysis can, on the one hand, test this empirical assumption and, in this way, become relevant for bioethics to understand and validate an ethical argument. For example, if philosophers refer to the climate in media debates to justify slippery-slope arguments, a distinct empirical analysis of this media debate can show whether this part of the argument holds. On the other hand, the empirical results can contribute as a premise to a new subsequent ethical argument.

Second, media debates allow an insight into the *applicability and the impact* of ethical arguments. Analysing media debates provides insights into the conditions and circumstances in which bioethical ideas, convictions, or arguments are deemed applicable or can become effective in practice. Thus, besides estimating the potential impact of certain bioethical ideas, media debate analyses can contribute to the practical applicability of ethics (see sections above on the societal and practical significance). If ethical considerations are supposed to unfold effects in the societal reality– as they usually should [24]– understanding the conditions and the social context in which ethical arguments are applied and have an impact is of ethical relevance. Analyses of media debates represent one way to gather such an understanding, e.g., on how ethical argumentations are (mis)interpreted in public and what fosters their acceptance or rejection [24].

Hence, these considerations show that – due to their societal, practical, and ethical significance – the analysis of media debates combines the different potentials of empirical research for bioethics that have been addressed in connection with the so-called empirical turn [22, 25]. However, these observations also underline the necessary methodological expertise. While some media debate analyses can be performed by social scientists, the description of ethical aspects and the identification and evaluation of moral problems require additional competence in ethics.

## Systematizing the landscape: rapid scoping review

To gain an initial representative understanding of how media debate analyses have been used and are useful for bioethics, as well as which methodological approaches were used to different ends, we conducted a rapid scoping review of the literature. This review offers a systematic and resource-efficient overview of studies analysing health-related media debates [26], highlighting the different methodologies applied in the field. It provides a broad perspective of the study landscape, helping to illustrate our methodological and theoretical considerations. As such, the rapid scoping review serves as a foundation for identifying methodological requirements and challenges and to develop perspectives for methodologically founded investigations of media debates in bioethics. Given the conceptual focus of this paper, an in-depth examination of individual studies that other review methodologies provide is beyond this paper’s scope. Instead, the rapid scoping review efficiently captures the research objectives and the methodological approaches necessary to underline our conceptual arguments in this paper.

### Methodology

**Fig. 1:**
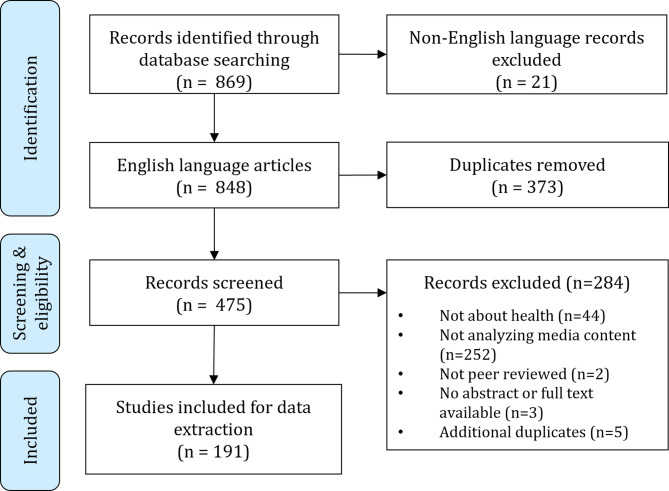
Flow chart illustrating the systematic publication selection process. Studies in languages other than English were excluded from this review

We systematically searched relevant articles in PubMed, Web of Science (Core Collection), and Scopus (see Table 1 for search algorithms and Fig. 1 for the article selection process). We included English-language articles that used an established methodology (e.g., qualitative or quantitative content analysis, thematic analysis) to analyse the content of traditional mass media or social media on a health topic (broadly defined, including One Health, public health, health research, and medical education) with a reference to bioethics (regarding topic, affiliation of authors, scope of journal etc.). Following our above-introduced definition of media debates, we excluded analyses of fictional content (e.g., assessment of child TV programs), social media analyses focusing on private conversations (e.g., closed Facebook groups) and studies that analysed the content of advertisements. Non-English-language studies were also not included.

**Table 1:** Search algorithms (search was performed in November 2023)

Pubmed^1^	((mass media[MeSH Terms]) OR (“newspaper*“[Title/Abstract]) OR (“online news“[Title/Abstract]) OR (“popular press“[Title/Abstract]) OR (“media coverage“[Title/Abstract]) OR (“news article*“[Title/Abstract]) OR (“media reporting“[Title/Abstract]) OR (“social media“[Title/Abstract]) OR (social media[MeSH Terms]))	AND
	((biomedical ethics[MeSH Terms]) OR (analysis, ethical[MeSH Terms]) OR (analyses, ethical[MeSH Terms]) OR (ethic*[Title/Abstract]) OR (moral*[Title/Abstract]) OR ethic*[Affiliation])	AND
	((“content analysis“[Title/Abstract]) OR (“thematic analysis“[Title/Abstract]))	^2^
Web of Science	(TS=(“mass media”) OR TS=(newspaper*) OR TS=(“online news”) OR TS=(“popular press”) OR TS=(“media coverage”) OR TS=(“news article*”) OR TS=(“media reporting”) OR TS=(“social media”))	AND
	(TS=(ethic*) OR TS=(moral*) OR WC=(Ethics) OR WC=(Medical Ethics) OR OG=(ethic*))	AND
	(TS=(“content analysis”) OR TS=(“thematic analysis”))	AND
	(TS=(*health*) OR TS=(medicine) OR TS=(medical*) OR TS=(clinic*) OR WC=(Public, Environmental & Occupational Health) OR WC=(Health Care Sciences & Services) OR WC=(Health Policy & Services))	
Scopus	(TITLE-ABS-KEY(“mass media”) OR TITLE-ABS-KEY(newspaper*) OR TITLE-ABS-KEY(“online news”) OR TITLE-ABS-KEY(“popular press”) OR TITLE-ABS-KEY(“media coverage”) OR TITLE-ABS-KEY(“news article”) OR TITLE-ABS-KEY(“media reporting”) OR TITLE-ABS-KEY(“social media”))	AND
	(TITLE-ABS-KEY(“content analysis”) OR TITLE-ABS-KEY(“thematic analysis”))	AND
	(TITLE-ABS-KEY(ethic*) OR TITLE-ABS-KEY(moral*)) OR AFFIL(ethic*)	AND
	(TITLE-ABS-KEY(*health*) OR TITLE-ABS-KEY(medicine) OR TITLE-ABS-KEY(medical*) OR TITLE-ABS-KEY(clinic*))	

Notes: ^1^ Since PubMed is a database that specifically covers biomedical literature, we omitted the fourth search category focusing on health topics. ^2^ Thematic analysis was used as a form of qualitative content analysis in the literature and hence added as a keyword in the search string

From the included publications, we collected the following data from titles, abstracts, and other publication meta-data: (1) research field of the periodical; (2) first author’s field of affiliation; (3) topic of the paper; (4) unit of analysis (e.g. social media posts, newspaper articles); (5) methodology to analyse media content (qualitative, quantitative, mixed methods, Machine Learning-based); (6) presence of data triangulation; and (7) the contribution to bioethics. No full-text analysis was conducted.

### Interdisciplinary landscape

**Fig. 2:**
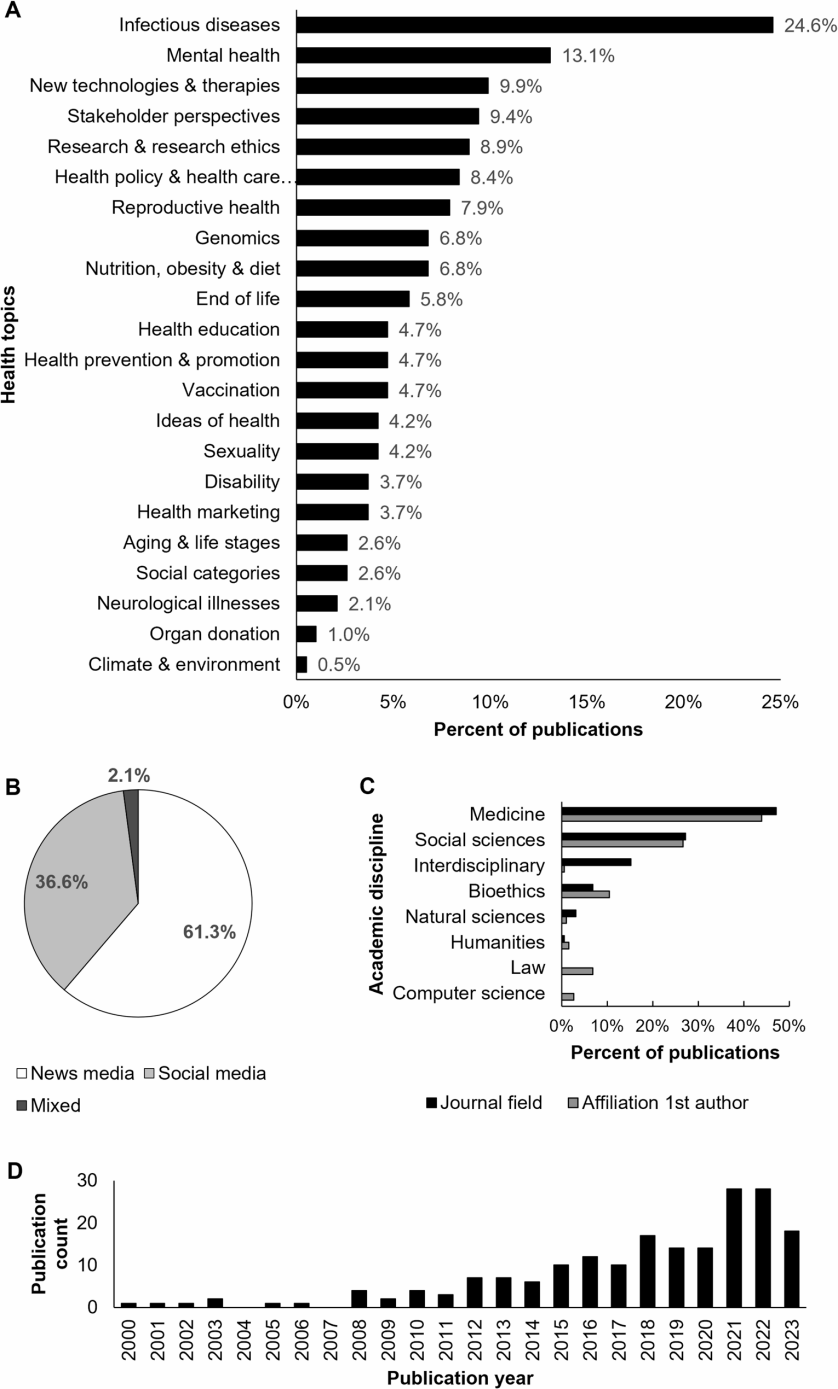
Characteristics of media debate analysis publications about health. (**A**) Topics covered. (**B**) Arena of debate. (**C**) Academic fields represented. (**D**) Publications per year

To assess the interdisciplinary landscape of the 191 included publications, we extracted information on topics, methodologies, and academic fields. Topics were identified by applying Philipp Mayring’s methodology of a summarizing content analysis, which includes a step-wise paraphrasing and abstraction of content [27]. The most frequent topics were public health-related, including infectious diseases (24.6% of articles, Fig. 2A), mental health (13.1%) as well as nutrition, obesity and diet (6.8%). Other frequent topics addressed technological innovation in healthcare, including new technologies & therapies (9.9%), reproductive health (7.9%), and genomics (6.8%). Further, topics addressing the health system, including stakeholder perspectives (9.4%), health research & research ethics (8.9%), as well as health policy & health care systems (8.4%) were addressed. Various other health topics were addressed, too. Figure 2B shows that most analyses were conducted based on debates in news media (61.3%). Figure 2C indicates the variety of academic fields represented in health-relevant media debate analyses. Medicine was the most-represented field, followed by the social sciences and bioethics. Moreover, 15.2% of the articles were published in interdisciplinary journals. Our rapid scoping review further shows that the number of relevant publications has steadily increased since 2008 (Fig. 2D).

### Contribution to bioethical inquiries

**Fig. 3:**
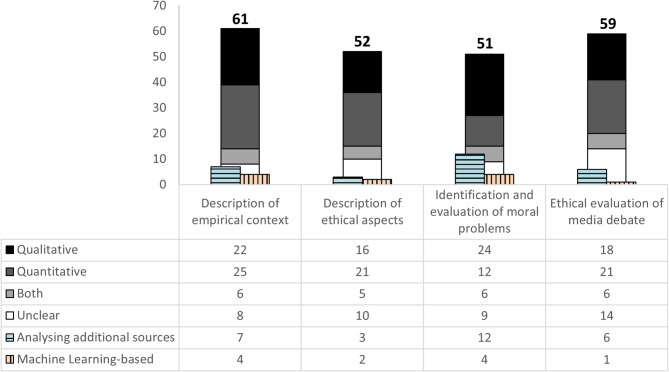
Categorization of media debate analyses regarding their contribution to bioethical inquiries

Based on the considerations on the threefold significance of public media debate analyses for bioethics and initial findings from the rapid scoping review, we defined four categories of how media debate analyses have contributed to bioethical inquiries so far (Fig. 3). They show the different content-related potentials of the investigation of media debates in bioethics as well as their methodological range. While we conceptualized the first three categories as mutually exclusive, the fourth category (ethical evaluation of media debate) was sometimes coded in addition to one of the other categories. In the following, we will describe these categories by giving illustrative examples and emphasizing characteristics as identified through the rapid scoping review.

#### Description of empirical context

Around one in three included publications (*n* = 61, 31.9%) did not engage explicitly in any ethical rationale or discussion but provided descriptive information on the content of media debates. Methodologically, qualitative and quantitative approaches were similarly represented. Additionally, four articles in this category used Machine Learning-based text categorization methodologies. For example, Indra and colleagues analysed social media posts about obesity, physical activity, and diets in the context of healthy lifestyles [28]. They performed sentiment analysis and topic modelling on these posts to identify the tone of the debate and the topics covered as determined by word frequencies, combinations, and relative proximities.

As argued above, even though they are descriptive, such investigations may still be of relevance for bioethical inquiries: They can provide an empirical basis for further bioethical analyses, even if no such analysis is provided in the publication itself. A pertinent example is a quantitative content analysis by Zimmermann and colleagues about the content, evaluations, and stakeholder influence in newspaper coverage of predictive genetic testing in Switzerland and the United Kingdom [29]. Contrasting their findings with other studies in the field of science communication, they showed how the debate fostered a more active public engagement with the topic than previous science communication topics and how the debate was much less salient in German-speaking Switzerland as compared to the United Kingdom.

#### Description of ethical aspects

As a second category, we identified 52 publications (27.2%) which explicitly described ethical aspects of health topics while not engaging in any more in-depth investigation and evaluation of ethical aspects. For example, Zimmermann and colleagues analysed what ethical issues pertinent in scholarly debates about predictive genetic testing were reported on in British newspapers. They quantified what ethical issues were particularly salient in newspaper reporting, identified what ethical issues were underrepresented, and discussed what ethical issues the public should be more explicitly informed about and why [30]. This allowed a detailed description of how ethical aspects within a specific context were represented in a pertinent media debate. In this way, this example illustrates the above-outlined significance of media debate analysis through a reflection of morality.

Further, such descriptions of ethical aspects covered in media debates touch upon the societal relevance of media debate analyses. For example, Chandler and colleagues qualitatively analysed online comments in response to news articles reporting on a Canadian patient who had been in a vegetative state for several years and was reported to communicate via fMRI neuroimaging [31]. The findings illustrated important moral deliberations in the online comments, including the quality of life of this patient, the question of withdrawing life support and options of the patient consenting to this, and the accuracy of diagnosing brain death. In concluding that “[t]hese public perspectives are relevant to the obligations of clinicians, lawyers, and public policymakers to patients, families, and the public” [31], the authors refer to the practical and ethical significance of their analysis.

Methodologically, publications in this category mostly followed traditional methodologies of media content analyses, including quantitative content analysis (*n* = 21, 40.4%) and qualitative content analysis (*n* = 16, 30.8%, see Fig. 3).

#### Identification and evaluation of moral problems

More than a quarter of the reviewed publications (*n* = 51, 26.7%) identified and evaluated moral problems regarding health-related topics in addition to describing the content of media debates. Such publications employed more qualitative (24/51, 47.1%) than quantitative procedures (12/51, 23.5%). Some 12 publications in this category (23.5%) analysed other data sources in addition to media content. This seems plausible since qualitative approaches and data triangulation offer more possibilities to examine a topic in-depth, and, in this course, to identify and evaluate ethical problems. For example, Rosenberg and colleagues analysed qualitative semi-structured interviews in addition to expert comments in Finnish media to demonstrate the social impact of the orphan drug Nusinersen [32]. By combining these two analyses, they outlined competing attitudes and values and showed that these may be classified differently depending on the situation.

As an example of a qualitative content analysis, Ross Arguedas investigated the media coverage of orthorexia nervosa [33]. Applying inductive thematic analysis, the author identified different framings of orthorexia but also a heterogeneous assignment of responsibility depending on the explanation of the causes of the eating disorder. Further, exemplifying a quantitative content analysis, Zimmermann and colleagues investigated solidarity and personal responsibility as important normative reference points in newspaper coverage in Germany and German-speaking Switzerland [34]. Using quantitative content analysis, they identified different contexts and areas of application of these two concepts. Finding that the limits of solidarity were only rarely mentioned in newspaper coverage about COVID-19, they called for a more distinct consideration of these limits to sustain solidarity as a resource in further crises.

#### Ethical evaluation of media debates

Finally, around one in three included publications (*n* = 59, 30.9%) discussed and evaluated the media debate itself from a normative perspective. For example, they identified blind spots, shortcomings, and tendencies in the media debate by focusing on how the media and the debate were embedded in a broader social and societal context. Such publications thus identified moral problems that arise or are reinforced *because of* the media debate.

For example, Aspler and colleagues conducted a content analysis of 286 articles from Canadian newspapers to investigate how the fetal alcohol spectrum disorder is presented in the media debate [35]. Starting from inductive coding, they identified six major themes. In discussing these findings, they focused especially on exaggeration and misinterpretation in the debate and highlighted the risks of harmful stereotypical beliefs about indigenous people, confusion about health choices during pregnancy, and the unhelpful fueling of debates about sensitive issues surrounding women’s choices.

Some 32 of the 59 publications in this category (59.2%) were additionally assigned to other categories. For example, the above-mentioned study by Aspler and colleagues also aims to identify and evaluate moral problems [35]. This is not surprising since a certain thematic focus is usually necessary for a substantial ethical evaluation of the media debate.

In terms of methodology, the investigations in this category are diverse. Quantitative and qualitative approaches are equally spread. For example, Bosch and Wasserman chose a quantitative approach to analyse the tabloid media coverage of COVID-19 in South Africa [36]. Starting from the proposition that tabloid newspapers are often criticized for their sensation- and entertainment-orientated journalism, they investigated 1050 online news stories in the tabloid *Daily Sun* and found that the majority of the coverage was topic-oriented and neutral. Thus, using a quantitative approach to the evaluation of media debate, they show how the societal and practical significance of media debates can be addressed in media debate analyses.

The study of Patterson and colleagues applied both qualitative and quantitative content analysis to investigate how media coverage in the UK represented ‘binge’ drinking [37]. They found a “disproportionate focus on women’s ‘binge’ drinking” and discussed the potential effects of reinforcing harmful gender stereotypes. Furthermore, they formulated concrete suggestions on how media framing could be improved by a more comprehensive media engagement of public health advocates. Hence, this investigation is a very significant example illustrating how the societal and practical significance of media debates can be addressed in a field with different methodological approaches and different concrete research questions.

## Towards systematic approaches to media debate analyses in bioethics

Media debate analyses are, as we have argued, of high relevance for empirically informed bioethics. Due to their societal, practical, and ethical significance, the investigation of media debates can provide insights for bioethics at the specific intersection of politics, society, healthcare, and ethics.

Our rapid scoping review shall provide a first overview of the field at this specific intersection and has limitations. First, it includes only articles published in English language with an abstract in peer reviewed journals listed in PubMed, Scopus, or Web of Science. Even though we strived to include all relevant synonyms for the search algorithms, the scope of included articles was limited to analyses of the content of media debates with an explicit connection to ethics (Table 1). Many country-specific media analysis debates might have been conducted and published in non-English language and/or alternative repositories (e.g., theses, reports). Due to the ethics keywords in the search algorithm, the number of articles describing empirical context are underestimated. Second, while we selected relevant articles and collected the data systematically (Fig. 1), no formal inter-coder reliability test was performed and data collection was purely based on titles and abstracts, not full texts.

Even though our rapid scoping review gives only a first impression of the landscape of media debate analyses in bioethics, its results show a *growing interest* in public discourses in general and media debates in particular for bioethics. They further reveal a considerable *heterogeneity of research interests* in the field, ranging from a focus on the political aspects of such debates to an emphasis on the practical effects of such discourses on concrete action in healthcare and medicine to specific ethical questions. Depending on the focus, these analyses speak to various levels of relevance – societal, practical, and ethical– as outlined above.

Finally, the review illustrates *methodological plurality*. This concerns the selection and sampling of the media (print, online, social media etc.), the significance of media analysis in the bioethical investigation itself, right up to the fundamental methodological question of how the analysis should be carried out in concrete terms. This observed methodological plurality may point to a methodological uncertainty regarding bioethical investigations of media debates, despite the massive methodological developments in bioethics since the “empirical turn” [38]. Fundamental methodological questions related to media debate analysis in bioethics, e.g., regarding the fruitful entanglement of empirical research and ethics, call for further methodological reflections in the face of these uncertainties. In the following section, we address the pertinent methodological issues and attempt to shed light on methodological cornerstones when conducting analyses of media debates for bioethics.

### Methodological cornerstones of media debate analyses in bioethics

As in any empirical inquiry, the study design and methodology of a media debate analysis must be adapted to a project’s purpose, aim, and context. Our rapid scoping review illustrated the vast plurality of possible approaches. In the following, we suggest four relevant methodological dimensions to be considered by bioethicists who seek to conduct media debate analyses for bioethics projects.

First, a study can be more *descriptive* (description of empirical context; description of ethical aspects) or focus more on *normative* perspectives (identification and evaluation of moral problems; ethical evaluation of media debate). As we have argued, both perspectives are necessary and valuable for bioethics. However, descriptive perspectives should also strive to allow for and ease a normative evaluation of bioethical issues. This speaks to the pertinent scholarly discussions of overcoming the strict dichotomy between empirical and normative bioethics [39]. It also underlines the function of bioethics as a normative endeavour that does not only describe how practices in medicine and healthcare (as well as pertinent public discourses) are but also argues how they should be.

Second, there are primarily studies that apply either *quantitative* or *qualitative* approaches. Depending on the specific research questions, quantitative, qualitative, and mixed-methods approaches that combine quantitative and qualitative aspects can be helpful for bioethics. Qualitative approaches are more explorative and may facilitate an in-depth understanding of underlying moral norms, values, and concepts. Quantitative, codebook-based approaches are more useful for comparing media debates in different contexts (e.g., over time or in different countries) [40] or for quantifying the relevance of pre-known ethically relevant aspects in the debate. New forms of Machine Learning-based content analyses that are based on Natural Language Processing allow for explorative statistical analyses where the normative analysis hinges upon its combination with other methodologies and a posthoc interpretation of these statistical connections [41].

Third, the approaches differ regarding their disciplinary focus. While some are theoretically and methodologically attributed to the *social sciences*, others stand in the tradition of *moral-philosophical* investigations. The social sciences, particularly the field of communication sciences, provide established methodologies of media debate analyses that help to systematize the analysis and to avoid example-picking or anecdotal evidence. Being competent in these approaches is crucial for empirical bioethicists to set a coherent empirical approach that is driven by a bioethical research question. However, philosophical and ethical competence is equally necessary, for example for the reflection and justification of moral values, norms, and judgments [23]. Thus, such research underlines the necessity of interdisciplinarity for bioethical research.

Fourth, the approaches differ in their positioning at the intersection of politics, society, healthcare, and ethics. While some focus more on the political implications, others tend to focus on the practical effects or the ethical aspects. However, to maximise their significance for bioethics, media debate analyses should have a *specific bioethical orientation in the investigation* in all stages of the research process, as in every empirically informed bioethical investigation [22, 23]. This demands certain theoretical considerations: On the one hand, the study rationale and the phenomenon under study must be of relevance for bioethics. On the other hand, the different steps of data analysis, interpretation of findings and discussion of results should be guided and informed by the pertinent bioethical debate.

### Limitations of media debate analyses for bioethics

Media debate analyses are subject to limitations that are relevant when considering their usefulness for bioethical inquiries. First, media debates do not directly mirror public opinion. While mass media are considered to reflect and shape public opinion, these associations are neither linear nor representative. It is not possible to make quantitative extrapolations on what people think about a topic or how media influence public opinion, since the effect of media coverage on public opinion– and vice versa– is highly complex, as decades of media effects studies have shown [12, 14].

Second, even though we argue for bioethics as a normative endeavour, normative conclusions cannot be reached purely based on the empirical findings from media debate analyses. As in every empirically informed bioethics project, researchers must consider the difference between is and ought and consider the approaches of how to combine empirical and normative aspects in empirically informed bioethics [e.g., 4].

A third limitation concerns the intersection of politics, society, healthcare, and ethics in which media debate analyses for bioethics operate. As we have illustrated, such analyses can provide important insights into the portrayal of ethical aspects or the salience of moral problems. Yet, moral aspects reflected in media debates are unlikely to fully represent all relevant aspects of a given topic or society. The nature and content of media debates are shaped by various factors, including news values that make journalists favour certain news over others [42], the economic pressure on mass media that favours reporting generating most reads, or political and societal stakeholders who pursue their interests by taking part in media debates. Additionally, journalists or media outlets might follow their own political agenda or ideological orientation that shapes their reporting. Therefore, media debates must not be confused with any ethical truth but demand another consideration of the respective social contexts, practices and structures. A comprehensive analysis of media debates should consider the underlying and entangled norms, political agendas, power structures and speaker positions [e.g., 43].

### Future research

To enhance the significance of media debate analyses for bioethics even more, future research should focus on at least four aspects. First, further arenas of public debates should be included in a methodologically reflected way. The focus has been shifting from mass media (especially newspaper) reporting to the investigation of social media debates. Yet, other arenas, such as parliamentary debates and releases of civil society organisations, could be considered, too. Such a more comprehensive approach to investigating public discourses with their different arenas yields additional potential for bioethics, even though it requires supplementary methodological expertise.

Second, recent advancements in Natural Language Processing (NLP) technologies allow for more technology-assisted and digital content analysis. While we can observe a growing interest in digitally mediated discourses (e.g., social media), the application of Machine Learning-based NLP methodologies remains scarce in our literature review. Still, research in this area is evolving rapidly and includes studies comparing NLP-generated codings with manual codings [44] or NLP-based early warning systems to detect misinformation [45, 46]. Further research will have to show how such methods can contribute to the bioethical investigations of media discourses to drive the empirical turn in the digital era [47].

Third, the specific ethical significance of media debates could be brought in more comprehensively. Existing analyses of media debates try to understand ethical norms, values, concepts, and principles in a specific area of application and focus on the moral-philosophical level throughout their analysis [e.g., 22]. Future media discourse analyses should be aware of and confident about what they can contribute to fundamental ethical questions and ethical theory-building.

Finally, media debate analyses may not only be relevant for bioethics but may also have a political dimension themselves. By analysing and evaluating media debates, bioethicists may participate in the political arena. In this context, it is important to reflect on this position and the political consequences of such research without neglecting scientific rigorousness. This also comprises the question of how bioethics can feed their findings into the media debates and, thus, initiate a deliberative process based on its findings.

## Conclusions

The outlined approaches, their implications, and their limitations underline the significance and potential of media debate analyses for empirically informed bioethics. Media debate analyses can contribute to contextualizing and reflecting bioethics socially and politically by describing how ethical issues are discussed in media debates; by rooting normative arguments into evidence-based premises; by explaining and discussing societal perspectives and political developments; and by contributing to practical recommendations on how to improve care, public health interventions, and communication to the public. Yet, to fully realise this potential at the unique intersection of politics, society, healthcare, and ethics, interdisciplinary research that integrates methodological and theoretical expertise from the social sciences with domain knowledge from bioethics is essential. Fostering interdisciplinary collaborations and training young scholars from diverse social science disciplines in bioethics (e.g., through PhD programs) can support this effort.

## Electronic supplementary material

Below is the link to the electronic supplementary material.

Supplementary Material 1

## Data Availability

Data is provided within the manuscript or supplementary information files.
